# How are reasons for encounter associated with influenza-like illness and acute respiratory infection diagnoses and interventions? A cohort study in eight Italian general practice populations

**DOI:** 10.1186/s12875-021-01519-4

**Published:** 2021-08-28

**Authors:** Nicola Buono, Michael Harris, Carmine Farinaro, Ferdinando Petrazzuoli, Angelo Cavicchi, Filippo D’Addio, Amedeo Scelsa, Baldassarre Mirra, Enrico Napolitano, Jean K. Soler

**Affiliations:** 1 Department of General Practice, ICPC Club Italia Via Roosevelt 4, 81100 Caserta, Italy; 2 Department for Health, University of Bath, Claverton Down, Bath, BA2 7AY UK; 3 Institute of Primary Health Care (BIHAM), University of Bern, Bern, Switzerland; 4 Center for Primary Health Care Research, Clinical Research Centre, Lund University, Malmö, Sweden; 5 Mediterranean Institute of Primary Care, Attard, Malta

**Keywords:** Respiratory infections, Influenza, Primary care, Symptom assessment

## Abstract

**Background:**

Influenza-like illness (ILI) and Acute Respiratory Infections (ARI) are a considerable health problem in Europe. Most diagnoses are made by family physicians (FPs) and based on symptoms and clinical signs rather than on diagnostic testing. The International Classification of Primary Care (ICPC) advocates that FPs record patients’ ‘Reasons for Encounters’ (RfEs) as they are presented to them.

This study analyses the association of patients’ RfEs with FPs’ diagnoses of ILI and ARI diagnoses and FPs’ management of those patients.

**Methods:**

Cohort study of practice populations. Over a 4-month period during the winter season 2013–14, eight FPs recorded ILI and ARI patients’ RfEs and how they were managed. FPs recorded details of their patients using the ICPC format, collecting data in an Episode of Care (EoC) structure.

**Results:**

There were 688 patients diagnosed as having ILI; between them they presented with a total of 2,153 RfEs, most commonly fever (79.7%), cough (59.7%) and pain (33.0%).

The 848 patients with ARI presented with a total of 1,647 RfEs, most commonly cough (50.4%), throat symptoms (25.9%) and fever (19.9%). For patients with ILI, 37.0% of actions were related to medication for respiratory symptoms; this figure was 38.4% for patients with ARI. FPs referred six patients to specialists or hospitals (0.39% of all patients diagnosed with ILI and ARI).

**Conclusions:**

In this study of patients with ILI and ARI, less than half received a prescription from their FPs, and the illnesses were mainly managed in primary care, with few patients’ needing referral. The ICPC classification allowed a standardised data collection system, providing documentary evidence of the management of those diseases.

**Supplementary Information:**

The online version contains supplementary material available at 10.1186/s12875-021-01519-4.

## Background

According to the European Influenza Surveillance Network (EISN) [1, 2], the diagnostic code ‘influenza-like Illness’ (ILI) is defined as all acute respiratory infections accompanied by influenza-like symptoms, i.e. sudden onset, fever, myalgia, and respiratory symptoms. This diagnosis is commonly used in primary care, as it is not feasible for family physicians (FPs) to confirm whether or not every person with these symptoms is truly infected with the influenza virus because of the cost of diagnostic testing, poor availability of testing, and the lack of sensitivity of most rapid tests. [3, 4]. In the EISN context, the diagnostic code ‘acute respiratory infection’ (ARI) has been defined as any infection involving the respiratory tract, with or without fever, which lasts 1–2 weeks [5, 6]. ILI and ARI syndromes are a considerable health problem in Europe [7, 8] and one of the most frequent causes of medical attendance, with high general practice consultation rates mainly during the winter season [9, 10]. In many countries FPs play a big role in influenza epidemics, and most patients with ILI are treated in primary care [11–13]. During the winter, the levels of ILI and ARI increase, causing an increase FPs’ workload [14].

Increasing health care information needs are being recognized all over the world. In order to deliver optimal health care, professionals need information about the epidemiological situation in their community, and use diagnostic tools based on patients’ reasons for encounters, and information on best practice for the diagnosis and subsequent interventions [15]. In this context, the International Classification of Primary Care (ICPC) allows the study of the key elements of the encounter in Family Medicine (FM): namely the patient’s reason/s for encounter (RfE), and the doctor’s intervention/s and diagnostic label.

The use of ICPC is recommended by the World Organisation of Family Doctors (WONCA), and is widely reported in the literature as the most appropriate tool for the collection of international FM data [15–17].

Documenting and coding patients’ RfEs, in addition to their diagnoses and interventions, can improve the quality of primary care data [18–21], and can be useful for epidemiological studies [18–20]. Studies that include documentation of patients’ RfEs have allowed investigation of the prior and posterior probabilities of a diagnosis, which can be helpful when a patient from a specified sex/age group presents with a specific symptom or complaint [16, 19].

While there are some published data on the epidemiology, natural history and resource utilization associated with influenza in the Italian family medicine setting [9, 14], those data were collected in free-text format. Using the RfEs in the ICPC format allows family physicians to better formulate their diagnoses and has been demonstrated to influence the subsequent interventions [16, 19, 22–25]. It also allows researchers to compare data collected in one region or country directly with that from another [15, 17].

During the winter period FPs see many patients who could have either ILI or ARI, and a comparison of how the patterns of RfEs compare between the two diagnoses could help them in their management decision-making. The aim of the study was therefore to describe which RfEs were most commonly associated with influenza-like illnesses and acute respiratory infections diagnoses in eight Italian FPs’ patient populations during the winter season 2013–14, and how they were associated with FPs’ management of those patients.

## Methods

### The international classification of primary care

In this study, the content of family practice is measured with the ICPC [15–17].

This classifies patient data and clinical activity in the domains of family practice and primary care. It allows classification of the patient’s RfE, the problems and diagnoses managed, the interventions, and the ordering of these data in an ‘Episode of Care’ (EoC) structure [15–17]. The ICPC has a biaxial structure and consists of 17 ‘chapters’, each divided into 7 ‘components’ (Additional file 1) [17]. The RfE is defined as an agreed statement of the reason(s) why a person enters the health care system and represents the demand for care by that person [16, 17]. An EoC is defined as a health problem from its first presentation by the patient to the family physician, until the completion of the last encounter for it. It encompasses all contact elements related to that health problem [17].

### Selection of the subjects

Italian family physicians who belonged to ‘ICPC Club Italia’, an organisation with 12 members that works on the introduction and development of the ICPC in Italy, were invited to take part in the study. During a 4-month period (December 2013 to March 2014), they collected data on patients that they diagnosed as either having an influenza-like illness (ICPC code R80) or a different acute respiratory infection (comprising ICPC codes H71 otitis media, R74 acute upper respiratory infection including rhinitis, rhino pharyngitis, pharyngitis, R75 sinusitis, R76 acute tonsillitis R77 acute laryngitis, R78 acute bronchitis and R81 pneumonia) [13, 14, 25, 26]. We used the ARI and ILI categories as they have been found to be a valid tool for monitoring frequently occurring respiratory diseases [5, 6, 14, 15, 26–28]. Participating FPs were asked to complete an electronic form (Additional file 2) in an EoC structure based on the ICPC classification. For each EoC, the form prompted FPs to give data on patients’ age and sex, RfEs, the number of encounters for that EoC, procedures, the method of each encounter (at the FP’s practice, by telephone, at the patient’s home), whether the diagnosis was an ILI or an ARI, and whether or not the patient had had a pre-season influenza vaccination. To assist them with their coding, participating FPs were issued with an Italian-language version of the abbreviated, two-page version of ICPC-2 [29]. Completed data collection forms were sent to two independent coordinator centres by email.

As this was neither an interventional nor an observational study on pharmacological treatment, in accordance with local regulations the approval of the ethical committee was not required.

### Analysis

For continuous variables, mean values were calculated, and for categorical variables, percentages were calculated. Adjusted odds ratios (OR) and 95% confidence intervals (CI) were calculated using logistic regression analysis to compare the likelihood of specific symptoms presenting in patients diagnosed with ILI with those diagnosed as having ARI, to compare the symptoms of patients who were given prescriptions related to the respiratory system, were given sick notes, and who requested a return visit. Statistical analysis was performed using Epi-Info v7.1.4 (Center for Disease Control and Prevention, Atlanta, USA).

## Results

The study took place in the Lombardia, Emilia Romagna, Campania, and Basilicata regions of Italy. Eight family physicians, with 10,808 patients on their practice lists between them, took part in the data collection. Their demographics are shown in Additional file 3. None of them were involved in any other influenza surveillance at the time of the study.

During the data collection period, 1,536 patients were coded as having either ILI or ARI. Of these patients, 688 (44.8%) were diagnosed as having ILI, and 848 (55.2%) as having ARI. The patient demographics are shown in Table 1. Of those diagnosed with ARI, 328 (38.7%) were coded as having ‘upper respiratory tract infection’, 168 (19.8%) as ‘acute bronchitis/bronchiolitis’, 158 (18.6%) as ‘acute laryngitis/tracheitis’, and 128 (15.1%) as ‘acute tonsillitis’ (Table 2).

**Table 1:** Patient demographics, RfE rates, consultation rates and pre-season influenza vaccination rates

	Patients with ILI	Patients with ARI
Number of EoCs	688	848
EoCs by sex (% of all EoCs for ILI or ARI)		
Male	357 (51.9)	405 (47.8)
Female	331 (48.1)	443 (52.2)
EoCs by age range (% of all EoCs for ILI or ARI)		
< 30	130 (18.9)	120 (14.2)
30–44	226 (32.8)	201 (23.7)
45–59	201 (29.2)	226 (26.7)
60–74	107 (15.6)	180 (21.2)
≥ 75	24 (3.5)	121 (14.3)
Number of RfEs	2,153	1,647
Mean RfEs per EoC	3.1	2.0
Site of consultation (% of all consultations for ILI or ARI)		
Family physician’s own practice	423 (57.1)	622 (63.9)
Home visit	161 (21.7)	166 (17.0)
Telephone	157 (21.2)	186 (19.1)
Total consultations	741	974
Mean consultations per EoC	1.1	1.1
Number of procedures/interventions	1,347	1,521
Mean procedures/interventions per EoC	2.0	1.8
Received pre-season influenza vaccination (% of all patients with ILI or ARI)	66 (9.6)	201 (23.7)

**Table 2:** ICPC rubrics used for patients diagnosed with ARI

Code	Label	Number of times code used (%)
R74	Upper respiratory tract infection, acute	328 (38.7)
R78	Acute bronchitis/bronchiolitis	168 (19.8)
R77	Acute laryngitis/tracheitis	158 (18.6)
R76	Acute tonsillitis	128 (15.1)
R75	Sinusitis	29 (3.4)
H71	Otitis media	25 (2.9)
R81	Pneumonia	12 (1.4)
Total		848 (100)

There were 741 consultations for ILI, giving a mean consultation rate of 1.1 consultations per EoC; 423 of these (57.1%) took place at FPs’ own practices, 161 (21.7%) at patient’s homes, and 157 (21.2%) by telephone (Table 1). Patients with ILI had 1,347 procedures/interventions in total, a mean of 2.0 interventions per EoC.

In comparison, over the same time period, there were 974 consultations for ARI, giving a mean consultation rate of 1.1 consultations per EoC; 622 of these (63.9%) took place at FPs’ practices, 166 (17.0%) at patent’s homes, and 186 (19.1%) by telephone. Patients with ARI had 1,521 procedures/interventions, a mean of 1.8 interventions per EoC.

**Fig. 1:**
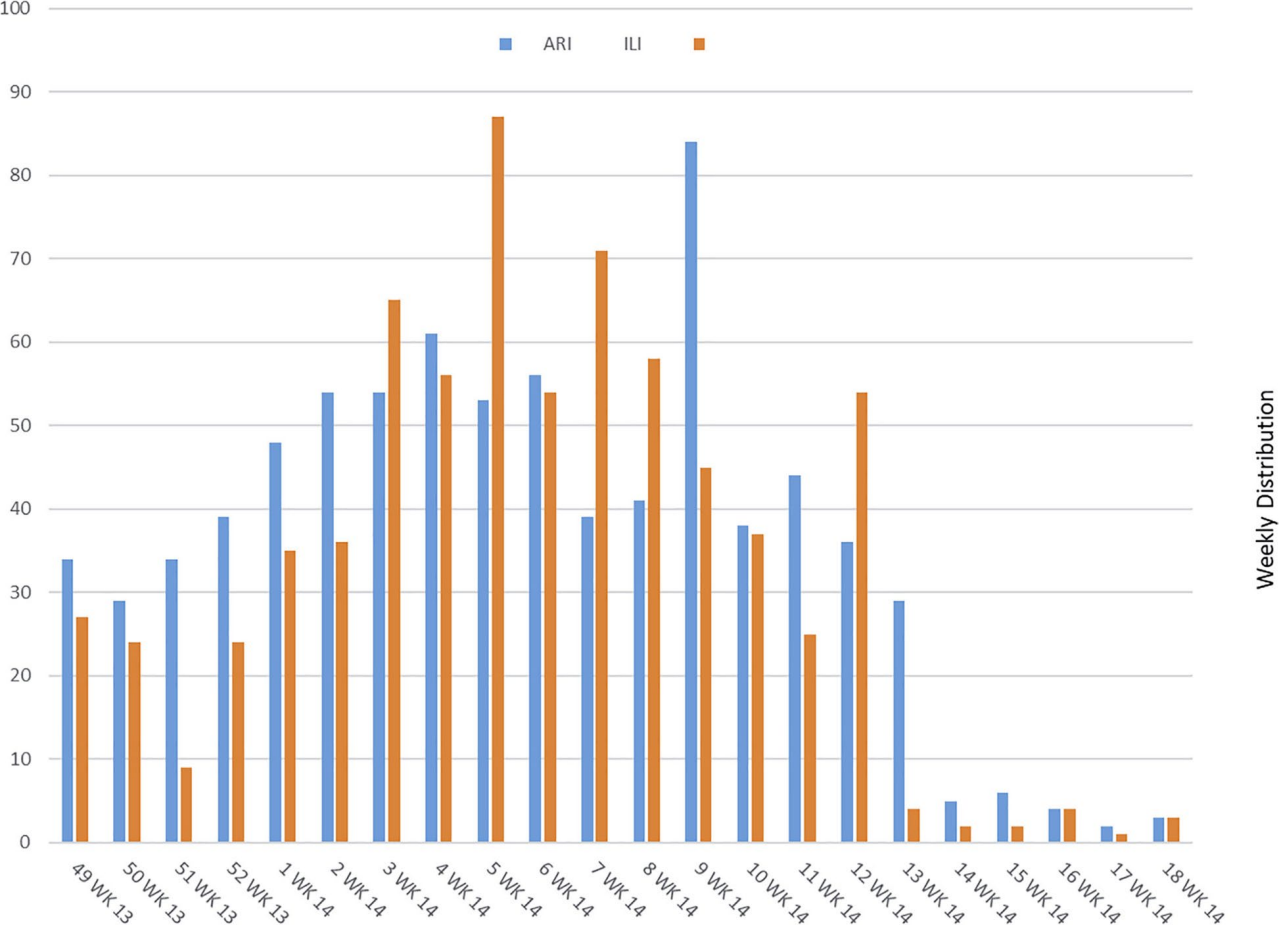
ILI and ARI weekly incidences during the study data collection period

For the first 6 weeks of the data collection period, considerably more patients were diagnosed with ARI than ILI (Fig. 1). In the final 5 weeks, few patients were diagnosed with either condition. There was no consistent pattern in between those times.

Between them, the patients presented with a total of 3,800 RfEs. Of the 2,153 RfEs recorded for patients diagnosed with ILI, the commonest RfEs were fever (79.7% of patients), cough (59.7%) and pain (33.0%). The patients with ARI presented with a total of 1,647 RfEs, most commonly cough (50.4% of patients), throat symptoms (25.9%) and fever (19.9%). Fever and pain were more likely in patients diagnosed with ILI than ARI (RR 4.0 and 8.0 respectively), while throat symptoms were less likely (RR 0.73) (Table 3).

**Table 3:** Commonest RfEs given by patients, and their frequencies for ILI and ARI. RfEs with low individual frequencies are grouped under ‘Other RfEs’

Code	Label	Number of ILI patients with this RfE (%)	Number of ARI patients with this RfE (%)	Risk ratio (95% CI)
R05	Cough	411 (59.7)	427 (50.4)	1.19 (1.08–1.30)
A03	Fever	548 (79.7)	169 (19.9)	4.00 (3.47–4.60)
R21	Throat symptoms	131 (19.0)	220 (25.9)	0.73 (0.61–0.89)
A01	Pain, general	227 (33.0)	35 (4.1)	7.99 (5.68–11.25)
R07	Chest pain	143 (20.8)	92 (10.8)	1.92 (1.50–2.44)
R50	Prescription request	79 (11.5)	58 (6.8)	1.68 (1.21–2.32)
R27	Fear of respiratory disease	58 (8.4)	45 (5.3)	1.59 (1.09–2.31)
R02	Shortness of breath	13 (1.9)	51 (6.0)	0.31 (0.17–0.57)
R23	Voice symptoms	14 (2.0)	47 (5.5)	0.37 (0.20–0.66)
R03	Wheezing	11 (1.6)	46 (5.4)	0.29 (0.15–0.56)
	Other RfEs	518 (75.2)	457 (53.9)	1.47 (1.32–1.64)

FPs referred six patients to specialists/hospital (0.39% of all patients diagnosed with ILI and ARI): two for A67 ‘general and unspecified problems’ (one for ILI and one for ARI), one for H67 ‘hearing problems’ due to ARI, one for K67 ‘heart complication’ due to ILI and two for R67 ‘respiratory complications’ due to ARI.

Table 4 shows the commonest actions undertaken by FPs for ILI and ARI. For patients with ILI, 37.0% of actions were related to medication for respiratory symptoms, and 25.2% were related to clinical examinations of patients’ respiratory symptoms. For patients with ARI, those figures were 38.4 and 35.4% respectively. In total, 464 of ILI patients (67.4%), and 632 of ARI patents (74.5%), had a clinical examination.

**Table 4:** Commonest procedures/interventions adopted by family physicians for patients with ILI and ARI

Code	Procedure	System for which action was taken	ILI (%)	ARI (%)
–50	Medication/prescription/request/renewal/injection			
R50		Respiratory	499 (37.0)	584 (38.4)
H50		Ear	36 (2.7)	7 (0.5)
A50		General and unspecified	32 (2.4)	12 (0.8)
D50		Digestive	10 (0.7)	6 (0.4)
F50		Eye	4 (0.3)	3 (0.2)
–3x	Medical examination/health evaluation^a^			
R3x		Respiratory	340 (25.2)	538 (35.4)
A3x		General and unspecified	79 (5.9)	64 (4.2)
H3x		Ear	30 (2.2)	23 (1.5)
D3x		Digestive	15 (1.1)	7 (0.5)
–62	Administrative procedures (sick notes)			
R62		Respiratory	124 (9.2)	111 (7.3)
A62		General and unspecified	36 (2.7)	7 (0.5)
–45	Observation/health education/advice/diet			
R45		Respiratory	42 (3.1)	72 (4.7)
D45		Digestive	13 (1.0)	5 (0.3)
A45		General and unspecified	12 (0.9)	3 (0.2)
–58	Therapeutic counselling/listening			
R58		Respiratory	31 (2.3)	52 (3.4)
–48	Clarification/discussion of patient's RfE/demand			
R48		Respiratory	30 (2.2)	12 (0.8)
–63	Follow-up encounter unspecified			
R63		Respiratory	4 (0.3)	11 (0.7)
–41	Diagnostic radiology/imaging			
R41		Respiratory	10 (0.7)	4 (0.3)
		Totals	1,347 (100)	1,521 (100)

^a^Combines sections 30 ‘Medical examination/health evaluation/complete’ and 31 ‘Medical examination/health evaluation/partial’

FPs requested diagnostic imaging related to the ICPC respiratory chapter in 0.7% of patients with ILI and in 0.3% of those with ARI.

The differences between the likelihood of ILI and ARI related to sex, age, and history of pre-season influenza vaccination were tested using logistic regression analysis (Table 5). Patient age and previous vaccination against influenza were significant predictors of ILI (age-group over 50 less likely to be affected by ILI than younger patients (OR 0.62, 95% CI 0.50–0.77); patients previously vaccinated against influenza less likely to be affected by ILI (OR 0.40, 95% CI 0.29–0.54). Sex was not a significant predictor.

**Table 5:** Logistic regression for difference in likelihood ILI and ARI with respect to sex, age and pre-season influenza vaccination

Variable	Odds ratio (95% CI)	*P* value
Age over 50	0.62 (0.50–0.77)	< 0.0001^*^
Sex (M/F)	1.16 (0.94–1.42)	0.15
Pre-season influenza vaccination (Yes/No)	0.40 (0.29–0.54)	< 0.0001^*^
Constant		0.48

^*^Significant at *P* < 0.05

In the logistic regression analysis to compare the likelihood of specific symptoms (Table 6), for patients with ILI given prescriptions related to the respiratory system, there was a significant association with symptoms of headache and generalised pain (OR 2.93, 95% CI 1.38–6.22 and OR 2.30, 95% CI 1.57–3.37 respectively). For patients with ARI, the commonest significant associations were with cough and throat symptoms (OR 2.49, 95% CI 1.73–3.67 and OR 2.40, 95% CI 1.57–3.68 respectively).

**Table 6:** Comparison of independent predictors for issuing a prescription, giving a sick note and requiring a return visit, for both ILI and ARI, calculated from a logistic regression analysis. For ease of interpretation, only symptom labels with a statistically significant OR are shown

	ILI				ARI			
	Code	Label	Odds ratio (95% CI)	*P* value	Code	Label	Odds ratio (95% CI)	*P* value
Prescriptions related to respiratory system	N01	Headache	2.93 (1.38–6.22)	0.005*	R05	Cough	2.49 (1.73–3.67)	0.001*
	A01	Pain, general	2.30 (1.57–3.37)	0.001*	R21	Throat symptoms	2.40 (1.57–3.68)	0.001*
Sick notes related to the respiratory system (R62)	N01	Headache	5.32 (2.47–11.4)	0.001*	A3	Fever	2.46 (1.27–4.78)	0.007*
	R05	Cough	1.70 (1.03–2.81)	0.03*				
Return visit	A03	Fever	127 (40–398)	0.001*	R05	Cough	22.1 (8.97–54-8)	0.001*
	R05	Cough	3.43 (1.78–6.61)	0.001*	A03	Fever	13.6 (6.64–28.1	0.001*
					R21	Throat symptoms	2.36 (1.11–5.03)	0.02*

^*^Significant at *P* < 0.05

The significantly associated symptoms for ILI patients who were given sick notes related to the respiratory system (R62) were headache (OR 5.32, 95% CI 2.47–11.4) and cough (OR 1.70, 95% CI 1.03–2.81). For ARI, fever was the only statistically significant association (OR 2.46, 95% CI 1.27–4.78).

During the data collection period, 168 patients (9%) requested a second visit for the same health problem, and 11 (0.7%) a third visit. For ILI, the main predictor of a return visit was fever (OR 127, 95% CI 40–398), followed by cough (OR 3.43, 95% CI 1.78–6.61). For ARI the predictors were cough, fever and throat symptoms (OR 22.1, 95% CI 8.97–54.8; OR 13.6, 95% CI 6.64–28.1; and OR 2.36, 95% CI 1.11–5.01 respectively).

## Discussion

### Principal findings

This study is the first investigation of the distributions of RfEs for ILI and ARI diagnoses made in an Italian primary care setting, collecting data on elements of doctor-patient encounters in an EoC structure. The commonest RfEs recorded for patients diagnosed with ILI were fever, cough and pain. Patients diagnosed with ARI presented most commonly with cough, throat symptoms and fever. Fever and pain were more likely in patients diagnosed with ILI than ARI, while throat symptoms were more likely in patients diagnosed with ARI. Less than half of all patients received a prescription, and fewer than 1% of patients were referred to specialists and/or hospitals or had tests requested. Subjects who had been vaccinated for influenza, and those aged over 50, were less likely to be diagnosed as having an influenza-like illness.

For patients who were given prescriptions, the symptoms tended to be different for ILI and ARI, with headache and generalised pain being commoner in patients who were subsequently diagnosed as having ILI, and cough and throat symptoms being more often seen in patients with ARI. For ILI fever and cough, and for ARI cough, fever and throat symptoms, were the main predictors of return visits for those patients.

### Comparisons with other literature

**Fig. 2:**
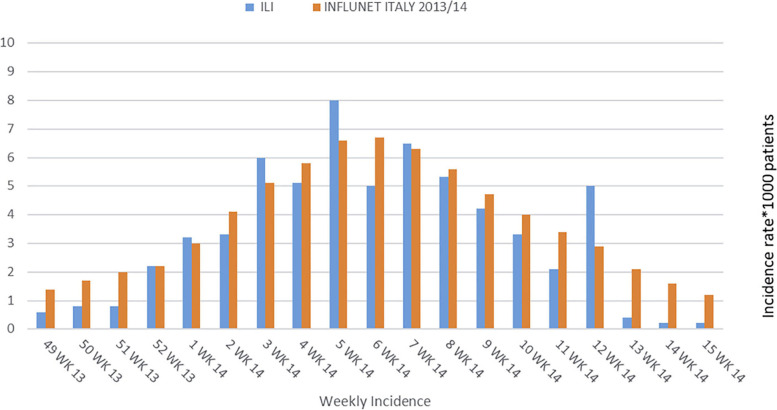
Weekly incidence of ILI in this study compared with the equivalent Italian influenza national sentinel surveillance (Influnet Italy) data for the same time period

Although the ILI patients in this study were not tested for presence of the influenza virus, the weekly ILI incidence recorded by this group is comparable to that of the Italian influenza national sentinel surveillance data (Influnet Italy) [30] for the same time period (Fig. 2). There were no comparable ARI Italian national surveillance data.

Our results are compatible with those of a multinational study which reported that, in individuals with ILI, the two best predictors of a laboratory-confirmed diagnosis of influenza were cough and fever. The authors included eight double-blind, placebo-controlled studies involving 231 study centres in North America, Europe, and the Southern Hemisphere. Of 3,744 subjects enrolled, 2,470 (66%, mean age, 35 years) were laboratory-confirmed to have influenza. Of those, 49.5% were females [31]. In our study, the mean age of ILI subjects was 44 years, and 48.1% were females. Despite a different methodology and study population, both studies confirm that, although FPs are often informally aware of the arrival of influenza virus in the community, their knowledge could be increased with the help of better surveillance and rapid confirmation of infection, especially at the start of an epidemic, when information is scanty. Since it is not feasible for FPs to collect diagnostic specimens from their patients during pandemic influenza [3, 4], the combination of better surveillance with the symptoms of cough and fever could improve the accuracy of FPs in making a clinical diagnosis of influenza.

In a cohort study that took place during the 1998/9 Italian winter epidemic period, 202 FDs performed almost 200,000 visits to 276,000 patients. A total of 6,057 cases of ILI were studied [9]. In contrast to our cohort, the most prevalent systemic symptoms were headache (70.2%) and myalgia/arthralgia (70%), followed by anorexia (59%) and feverishness (35.4%). The most prevalent respiratory symptoms were cough (82%) and sore throat (62.8%). Compared with our data, a much higher proportion of that group of patients received at least one prescription (97.3%), while a similar proportion had received pre-season vaccinations for influenza (5.9%). A higher proportion (4.2%) of that group needed a diagnostic test, specialist assessment or hospitalisation. Many more of the 1989/9 cohort were seen at a home visit (65.7%), but no telephone consultations were recorded.

Another study has shown that linking antibiotic prescriptions to specific diagnoses using the International Classification of Disease and Related Health Problems – Tenth Revision (ICD-10) could reduce those prescriptions [32]. Since FPs are responsible for most antibiotics prescribed to humans, a more specific ability to diagnose different respiratory infections, using both defined FM tools to collect data and appropriate guidelines, may help them better manage these drugs.

In a prospective observational study involving 2,191 ILI and ARI patients (49.8% females) that took place during the 2003/4 Italian winter influenza epidemic period, 508 cases of ILI and 1,683 ARI were gathered [14]. Compared to our population, in that study the percentage of ILI-ARI subjects was higher in the age ranges 5–14 and 45–65, and lower in patients over 65. While one may have expected fewer home visits in that study due to its younger population, 30% of those patients, higher than the 17% in our study.

A cross-sectional study on the ability of 60 primary care physicians to diagnose respiratory diseases found that, out of 235 patients (65.5% females) diagnosed as having ARI [33], the most prevalent respiratory symptoms were cough (90%), followed by fever (50%) and dyspnoea (25%). In that study, FPs were invited to fill out a symptom-based standardised respiratory questionnaire. Their results indicate the highest agreement between the diagnoses of the FPs and the respiratory physicians in ARI (k. 0.53,95% CI 0.46–0.60). Despite a different population and methodology, our study reached a similar conclusion in managing these diseases, with fewer or no referrals to specialists.

In a year-long retrospective study of 439 patients (71% females) seen in primary care because of ARI, the most common symptoms were found to be cough (present in 64% of ARI patients seen), sore throat (55%) and nasal symptoms (47%). FPs ordered rapid testing for group A streptococci in 18% of patients and chest x-rays in 8% of them. Twenty patients were referred to specialist. Clinicians prescribed antibiotics in 213 (49%) of them [34]. The authors concluded that interventions like accurate, reliable pre-visit triage and management, and internet-based medical visits, or E-Visits, which reduce ARI visits, have the potential to decrease inappropriate antibiotic prescribing, reduce the burden of ARI office visits on the health care system and offer more convenience for patients [34, 35]. In our cohort, the percentage of all respiratory prescriptions for 848 ARI patients was 38.8%. FPs referred four patients to specialists/hospital and 632 of them (74.5%), had a clinical examination. It may be that the recording of the patient’s reason for encounter, as well as the doctor’s diagnosis, may have triggered a more appropriate management in our patients. The ICPC is a classification which allows precise ordering of the data elements and concepts within a domain, with unique codes for unique and defined concepts [15, 17].

In a recent prospective study in Denmark, 2,323 ARI patients were diagnosed with either acute pharyngotonsillitis, acute otitis media, acute rhinosinusitis, acute bronchitis, pneumonia or acute exacerbation of COPD, according to the second edition of International Classification of Primary Care (ICPC-2). Less than half of all patients diagnosed with ARI received a prescription, which is lower than the antibiotic prescribing rate for a variety of ARIs in a recent study in Denmark [32]. Their conclusions ‘to improve antibiotic prescribing in general practice, it is important to focus on both the diagnostic process and the prescribing patterns’, are similar to ours.

In our study, some of the patients’ reason for encounters were independent predictors of issuing a prescription in both diseases. This is in accordance with other studies that showed a strong association between patients’ RfEs and the interventions made by their FPs [16, 19, 22, 24].

### Strengths and weaknesses of the study

The study used an internationally validated tool to collect data in general practice [15, 17, 22, 36, 37] on aspects of doctor-patient encounters in an EoC structure, over a complete winter influenza epidemic period. The age and sex profile of the cohort was similar to that of the Italian population as a whole (Additional file 4). The data were collected from patients’ electronic medical records, and many studies suggest that these data do not differ significantly from survey data based on self-report [16, 20, 38, 39].

Of the 1,536 patients whose data were used in the study, 36 were diagnosed as having both ILI and ARI. However, this is unlikely to have affected the analysis, as in each of those patients their ILI and ARI episodes of care were over different time-periods.

We had fewer participating doctors than in some other studies of ILI in primary care [9, 33], and we used a convenience sample of FPs, so our findings may not be generalisable to other Italian FPs. None of the patients diagnosed with ILI had laboratory testing for the influenza virus so some may not have been suffering from influenza. However, this reflects normal clinical practice and is compatible with EISN standards [1, 2], and the weekly incidence profile of ILI in our group was similar to that of national Italian influenza diagnoses over the same time period. Because FPs did not code the EoCs of all the patients that consulted them during the data-collection period, an estimation of the predictive value of symptoms for ILI and ARI diagnoses was not possible.

In Italy 67% of FPs work in an urban area [40, 41], and in our study half of the FPs were rural. At the time of the study, females made up 51.5% of the Italian female population [42], closely comparable with the 51.8% of the population of the participating practices in our study who were female (Additional file 4). In Italy, all citizens are registered with a primary care doctor, so the practice populations represent a cross-section of their local population. The ages of the participating practice populations closely aligned to those of the Italian population, with exception of patients were aged below 20 who were under-represented. This is likely to be because, in Italy, patients aged under 14 years are mainly seen by paediatricians.

The ICPC coding was done by physicians during routine clinical practice, so there may have been some omissions in RfEs, diagnoses and process codes due to individual errors. In addition, FPs may have seen, but failed to code, some ILI or ARI patients. The study was carried out in four out of the 20 Italian regions, with eight FPs. Their patients may be not representative of all the Italian population, so the findings may not be generalisable to that population. Though the study gathered information on ICPC Component 3 (medication, treatment and procedures), participating doctors were not asked to record the kind of medication they prescribed.

### Implications

Patients who have ILI and ARI can be managed with very low levels of diagnostic testing and specialist referral. The lower levels of ILI in patients aged over 50 may be due to the higher rate of pre-season influenza vaccination in that group. Primary care clinicians in other geographical areas will be able to compare these data with their own activity by using the ICPC coding system.

Knowledge of how the predictors for ILI and ARI compare will help doctors to implement early infection-control strategies and to assess the appropriateness of drug therapy.

## Conclusions

This study describes the RfEs that were most commonly associated with influenza-like and acute respiratory infections syndromes in eight Italian practice populations. Less than half of all patients diagnosed with ILI received a prescription, and the illness was managed almost entirely in primary care with very few patients referred to a specialist or for a test. Using the ICPC classification allowed a standardised data collection system, providing documentary evidence of the management of those diseases.

## Supplementary Information

**Additional file 1.** ICPC Structure.

**Additional file 2.** Electronic form built in an EoC structure, based on the ICPC classification, and used to collect the data.

**Additional file 3.** Demographics of participating family physicians.

**Additional file 4.** Age and sex profiles of the participating practice populations compared with the Italian.

## Data Availability

The dataset supporting the conclusions of this article is available in the Zenodo repository [unique persistent identifier and hyperlink to dataset] https://zenodo.org/record/47336#.VuGgzuao0g5%5D.

## Abbreviations

ILI: Influenza-like illness
ARI: Acute respiratory infections
FPs: Family physicians
ICPC: International Classification of Primary Care
RfEs: Reasons for encounters
EoC: Episode of Care
EISN: European Influenza Surveillance Network
R80: Influenza
H71: Otitis media
R74: Acute upper respiratory infection
R75: Sinusitis
R76: Acute tonsillitis
R77: Acute laryngitis
R78: Acute bronchitis
R81: Pneumonia
OR: Odds ratio
CI: Confidence interval
