# Supplementary figures and images for: How are reasons for encounter associated with influenza-like illness and acute respiratory infection diagnoses and interventions? A cohort study in eight Italian general practice populations
Source: BMC Fam Pract. 2021 Aug 28;22:172. doi: 10.1186/s12875-021-01519-4 (PMC8401359; doi:10.1186/s12875-021-01519-4)

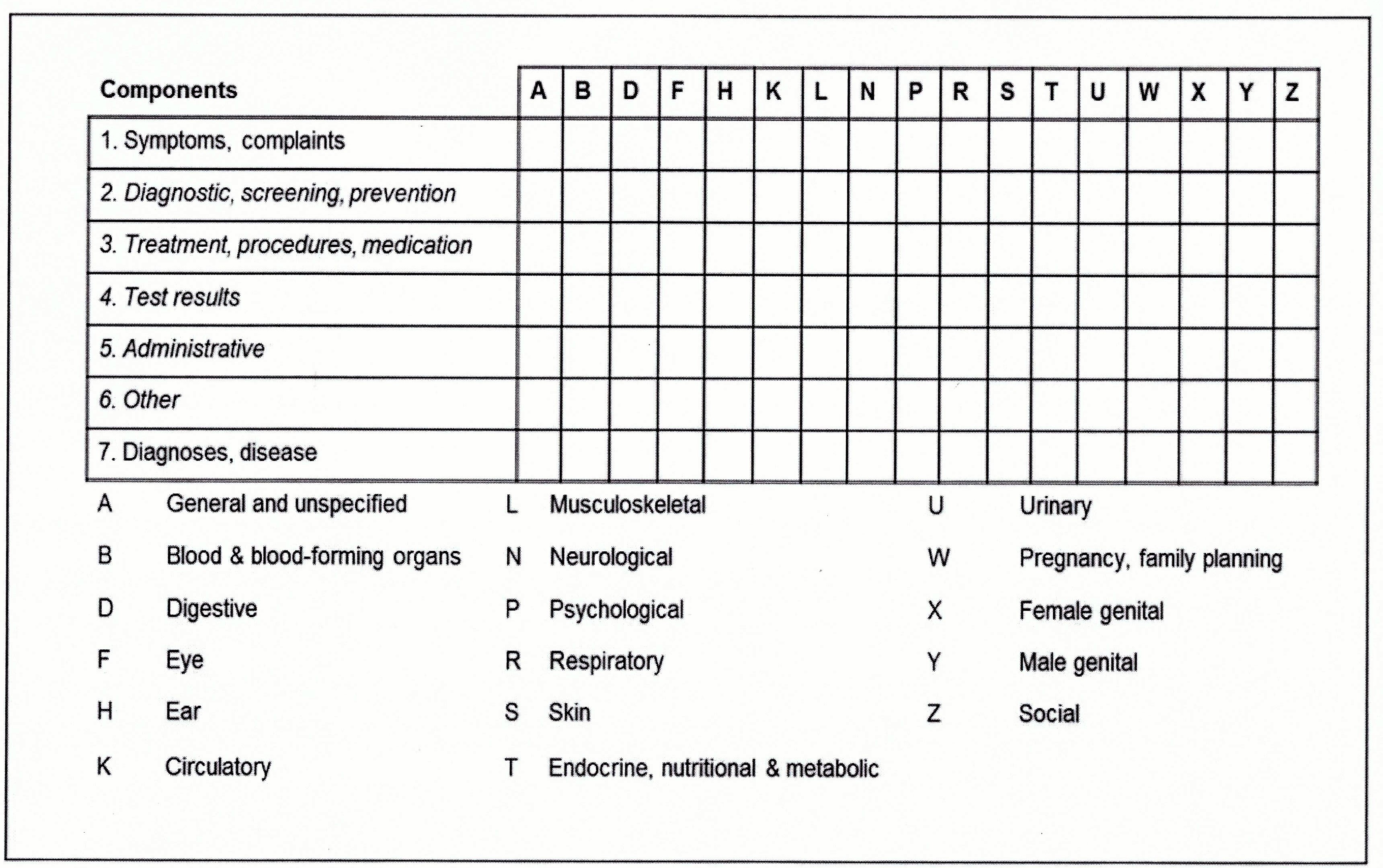

Supplement: Supplementary file 1 — Additional file 1. ICPC Structure. [file 12875_2021_1519_MOESM1_ESM.jpg]
