# Supplementary material for: How are reasons for encounter associated with influenza-like illness and acute respiratory infection diagnoses and interventions? A cohort study in eight Italian general practice populations
Source: BMC Fam Pract. 2021 Aug 28;22:172. doi: 10.1186/s12875-021-01519-4 (PMC8401359; doi:10.1186/s12875-021-01519-4)
Supplement: Supplementary file 2 — Additional file 2. Electronic form built in an EoC structure, based on the ICPC classification, and used to collect the data. [file 12875_2021_1519_MOESM2_ESM.pdf]

**2013-2014 Seasonal influenza-like illness and acute respiratory illness in eight Italian family practice populations.**

---

Date of first encounter Patient

Encounter Age Sex Flu Vaccination

RfE

Procedures

Diagnoses Chronic Diseases

**Save**

Date of second encounter Encounter

RfE

Procedures

Diagnoses

Date of third encounter Encounter

RfE

Procedures

Diagnoses

**Reset** **Save**
