# Supplementary material for: How are reasons for encounter associated with influenza-like illness and acute respiratory infection diagnoses and interventions? A cohort study in eight Italian general practice populations
Source: BMC Fam Pract. 2021 Aug 28;22:172. doi: 10.1186/s12875-021-01519-4 (PMC8401359; doi:10.1186/s12875-021-01519-4)
Supplement: Supplementary file 3 — Additional file 3. Demographics of participating family physicians. [file 12875_2021_1519_MOESM3_ESM.docx]

**Additional file 3.** Demographics of participating family physicians.

| Doctor code | Geographical region | Type of practice (Urban/ Rural/ Mixed) | Practice list size | Number of patients diagnosed with ILI during study | Number of patients diagnosed with ARI during study |
| --- | --- | --- | --- | --- | --- |
| 1 | Emilia Romagna | Urban | 1,534 | 73 | 214 |
| 2 | Emilia Romagna | Rural | 1,549 | 94 | 119 |
| 3 | Campania | Rural | 1,453 | 102 | 70 |
| 4 | Campania | Urban | 1,094 | 87 | 46 |
| 5 | Campania | Urban | 1,559 | 77 | 177 |
| 6 | Basilicata | Rural | 1,058 | 58 | 75 |
| 7 | Lombardia | Urban | 1,565 | 103 | 122 |
| 8 | Emilia Romagna | Rural | 996 | 74 | 25 |
