# Supplementary material for: How are reasons for encounter associated with influenza-like illness and acute respiratory infection diagnoses and interventions? A cohort study in eight Italian general practice populations
Source: BMC Fam Pract. 2021 Aug 28;22:172. doi: 10.1186/s12875-021-01519-4 (PMC8401359; doi:10.1186/s12875-021-01519-4)
Supplement: Supplementary file 4 — Additional file 4. Age and sex profiles of the participating practice populations compared with the Italian. [file 12875_2021_1519_MOESM4_ESM.docx]

**Additional file 4**. Age and sex profiles of the participating practice populations compared with the Italian population.

| Age | Population of participating practices (%) | Population of Italy  (%) |  |  |
| --- | --- | --- | --- | --- |
| Below 20 | 1,571 (14.5) | 11,310,660 (18.6) |  |  |
| 20-39 | 3,276 (30.3) | 14,532,892 (23.9) |  |  |
| 40-59 | 3,036 (28.1) | 18,293,135 (30.1) |  |  |
| 60-79 | 2,234 (20.7) | 12,768,555 (21.0) |  |  |
| 80 and over | 691 (6.4) | 3,877,426 (6.4) |  |  |
|  |  |  |  |  |
| Sex |  |  |  |  |
| Males | 5,210 (48.2) | 29,484,564 (48.5) |  |  |
| Females | 5,598 (51.8) | 31,298,104 (51.5) |  |  |
|  |  |  |  |  |
| Total | 10,808 (100) | 60,782,668 (100) |  |  |
